# Vestibular Disorders after Stapedial Surgery in Patients with Otosclerosis

**DOI:** 10.1155/2016/6830648

**Published:** 2016-01-19

**Authors:** Ditza de Vilhena, Inês Gambôa, Delfim Duarte, Gustavo Lopes

**Affiliations:** Department of Otolaryngology and Head & Neck Surgery, Hospital Pedro Hispano, Matosinhos, 4150-800 Porto, Portugal

## Abstract

*Introduction and Objectives*. Vertigo is a described complication of stapedial surgery. Many studies have been conducted to assess the improvement of hearing loss, but there are few studies that assess vestibular function after stapedial surgery. The aim of this study was to evaluate the presence and characterize the vertigo after stapedial surgery.* Methods*. We conducted a prospective observational study. Patients undergoing stapedial surgery in our hospital between October 2013 and December 2014 were invited to participate. The vertigo was assessed before and 4 months after surgery, using the Dizziness Handicap Inventory.* Results*. We included 140 patients in the study. 12 patients (8.6%) reported vertigo before surgery, and all of them denied vertigo after surgery. 36 patients (25.7%) reported vertigo four months after surgery, and none of them had vertigo before surgery. Postoperative total scores in patients with vertigo ranged between 2 and 18 points.* Conclusion*. The study shows that vestibular disorders may remain after the immediate postoperative period and reinforces the need for clarification of the patient in the informed consent act.

## 1. Introduction

Otosclerosis was first described in 1741 by Valsalva, who noted ankylosis of the stapes in an autopsy on a deaf patient and in 1894 Politzer defined otosclerosis as a clinical condition. It is an illness that affects the optic capsule, which forms part of the group of osteodystrophies, caused by changes in bone metabolism. It is characterised by the presence of several spots of reabsorption and bone repositioning. Despite significant research having been undertaken on the topic, the cause of otosclerosis remains unknown, although most authors consider it a multifactorial disease. There would appear to be a genetic predisposition with autosomal dominant inheritance, although it may occur sporadically.

Caucasians are most typically affected, although the prevalence rate of clinical otosclerosis is lower than 1% [1–3]. It is most common amongst the middle aged, with women most commonly affected [4–7]. Otosclerosis is a bilateral condition in approximately 60 to 80% of cases^6^.

It is one of the most common causes of conductive hearing loss and tends to be progressive in nature.

Treatment tends to be medical or surgical, with surgery being the preferred option, namely, a stapedotomy or stapedectomy. Although uncommon, in certain circumstances, some health professionals treat the condition medically with bisphosphonates or fluorine compounds, normally during the initial stages of the illness [8], either separate from or in combination with calcium and vitamin D [9]. The use of glucocorticoids has also been proposed when there is an associated sensorineural component [9, 10], and even the use of calcitonin has been suggested [11].

Dizziness is a complication of stapedial surgery mentioned in the literature, as it poses a significant risk of vestibular damage, perilymphatic fistula and prosthesis displacement, in addition to other complications [12, 13].

Various studies have been carried out to assess the improvement in conductive hearing loss or sensorineural loss after stapedial surgery; however, little research has been undertaken to assess vestibular functions after surgery.

Symptoms related to vestibular function disorders in patients with otosclerosis have also been documented prior to surgery [14, 15], with clinical evidence demonstrating the presence of dizziness in almost 17 to 23% of otosclerosis patients [15, 16]. The purpose of this study is to assess this presence and distinguish between dizziness before and dizziness after surgery in patients with otosclerosis.

## 2. Methods

We have designed a prospective and observational study, which was approved by the ethics committee at our hospital. All patients on whom stapedial surgery was carried out at our hospital between October 2013 and December 2014 were invited to participate.

As part of the service, partial posterior stapedotomy/stapedectomy are carried out under general anesthetic by employing a transcanal approach. By means of a horizontal incision in the skin of the outer ear canal's posterior superior wall, 5-6 mm from the annulus, a tympanomeatal flap is created that makes it possible to access the tympanic cavity. To guarantee better control of the oval window, curettage or drilling is performed several times in the canal's posterior superior wall, sparing the tympanic chord. After confirming the security of the stapes, the platinotomy, incudostapedial joint disarticulation, sectioning of the stapes muscle tendon, fracturing of the stapes superstructure, lengthening of the platinotomy/platinectomy, and installation of the Causse-type polytetrafluoroethylene prosthesis (Teflon) are carried out. For stapedial surgery, we accepted the recommendation of an air-bone gap equal to or greater than 30 dB.

The presence of dizziness was assessed before and 4 months after surgery. For instances of dizziness, the Portuguese version of the Dizziness Handicap Inventory questionnaire was used. The questionnaire comprises 25 simple questions, grouped into three categories: physical, emotional, and functional, with a variable score of between 0 and 100. Furthermore, the following data was collected: age, gender, and laterality. Patients submitted to revision surgery and those that failed to attend follow-ups were excluded. For the purposes of statistical analysis, the SPSS program was used and a level of statistical significance of *P* < 0.05 was allocated.

## 3. Results

140 patients were included in the study, 96 of whom (68.6%) were women. Age varied between 23 and 66, with an average age of 42. In regard to the ear operated on, right ear was for 91 patients (65%) and left ear was for 49 patients (35%). 11 patients (7.9%) showed signs of a bilateral illness.

48 patients (34.3%) mentioned dizziness in one of the two study periods. Before surgery, when questioned on the presence of any kind of vestibular disorder, 12 patients (8.6%) mentioned dizziness.

4 months after surgery, none of these patients mentioned any dizziness. 36 patients (25.7%) mentioned dizziness 4 months after surgery, of whom none had mentioned dizziness before surgery. 28.1% of the patients, without dizziness before surgery, developed vestibular complains after surgery. 92 patients (65.7%) did not mention any dizziness before or after surgery. These numbers are resumed at Tables 1 and 2. The total postoperation scores of the questionnaire on patients with dizziness varied between 2 and 18 points, with an average of 10 points. The most affected categories were physical and functional. There was no significant statistical difference between gender and age and the presence or seriousness of dizziness, whether in the presurgical period or in the 4 months after surgery.

## 4. Discussion

Women suffer otosclerosis more frequently than men, with a variable ratio of women to men of up to 2 : 1 [3]. Amongst patients subject to stapedial surgery at our hospital, during the study period, 96 (68.4%) were women and 44 (31.4%) were men. This data is similar to the figures obtained by other authors: 68.4% women in a study carried out in Spain [17] and 67% women in a French study [18]. We obtained a ratio of women to men of 2 : 1, which is also consistent with the literature [3, 17, 18].

Age varied between 23 and 66, with an average age of 42. The most affected age range was between 40 and 49 years of age (56%). In a study carried out on 475 Spanish patients, the most affected age range was between 15 and 45 years of age (62.2%) [17] whereas, as part of a study carried out in England on 65 English patients, the most affected age range was between 40 and 49 years of age [19]. Therefore, our results with regard to the most affected age range align with the results of publications released by other authors in the literature.

The condition was bilateral for 11 patients (7.9%) as part of our study, somewhat lower than in most of the studies published. As part of a study carried out in India, the condition was bilateral for 70% of study patients [20], whereas in an Iranian study this figure stood at just 18.2% [21]. The cause of a lower value having been recorded may be attributable to the fact that patients with previous stapedial surgery were excluded from the study.

As part of this study, when asked regarding the presence of any kind of vestibular disorder before surgery, 12 patients (8.6%) mentioned dizziness. This amount is lower than what is provided in the literature, with most studies returning dizziness rates of ranges between 17% and 23% [15, 16], for patients with otosclerosis. Amongst these patients, dizziness reduced after surgery, a phenomenon also identified by other authors [22]. The likely mechanism suggested by other authors is the fact that the quick recession is attributable to the sudden ruptures in the membranous labyrinth that cause changes in intralabyrinthine pressure due to changes in the volume of inner ear fluids. After stapedial surgery, as a result of movement in the ossicles and due to the prosthesis, a compensatory mechanism is established that prevents changes in pressure, which could explain the disappearance of dizziness.

As part of this study, in the 4th month following surgery, 36 patients (25.7%) reported dizziness. Dizziness is a common case in the days following surgery on the stapes [23, 24]; however, it rarely lasts longer than a week. Generally speaking, patients affected develop a permanent vestibular hypofunction, and over time they get used to the condition and are asymptomatic [25]. Birch and Elbrond reported a rate of dizziness lasting longer than one week of 4% [25], and Plaza Mayor recently reported a rate of persistent vertigo lasting 12 months of 2.6% [26]. The results obtained from this study were higher than those reported by the above authors, but very few studies assessing the presence of dizziness in the months following stapedial surgery have been carried out. Amongst these patients, as part of our study, all denied experiencing dizziness before surgery, which has made it possible to conclude that stapedial surgery could cause new vestibular disorders that are not related to the condition as such, which would remain in place after a 4-month period.

As part of this study, the total questionnaire scores in the postoperating period for patients with dizziness varied between 2 and 18 points, with an average score of 10 points; thus, dizziness can be considered light (whenever the result of the questionnaire is <30), with no significant effect on day-to-day activities.

## 5. Conclusion

Our study demonstrates that vestibular disorders may persist after the immediate postoperating period and highlights the need for patient clarifications at the time of providing informed consent.

## Figures and Tables

**Table 1:** Number and percentage of patients with and without dizziness, before and after surgery.

	Before surgery	4 months after surgery
	(*n* (%))	(*n* (%))
Dizziness	12 (8.6%)	36 (25.7%)
No dizziness	128 (91.4%)	104 (74.3%)

**Table 2:** Evolution of the two groups of patients (with and without dizziness before surgery), after the surgery.

Before surgery	4 months after surgery
Group of patients with dizziness (*n* = 12)	(i) All without dizziness

Group of patients without dizziness (*n* = 128)	(ii) 36 with dizziness (28.1%)
	(iii) 92 without dizziness (71.9%)
